# Cross-sectional association between 24-hour movement guidelines and depressive symptoms in Chinese university students

**DOI:** 10.7717/peerj.17217

**Published:** 2024-04-15

**Authors:** Yanqing Zhang, Xinli Chi, Liuyue Huang, Xingyi Yang, Sitong Chen

**Affiliations:** 1 Department of Physical Education, Xi’an University of Science and Technology, Xi’an, China; 2 School of Psychology, Shenzhen University, Shenzhen, China; 3 Department of Psychology, Faculty of Social Science, University of Macau, Macau, China; 4 School of Physical Education, Shanghai University of Sport, Shanghai, China; 5 Centre for Mental Health, Shenzhen University, Shenzhen University, Shenzhen, China

**Keywords:** Depression, Young adults, Physical activity, Sedentary behavior, Sleep

## Abstract

**Background:**

The world’s first 24-h movement guidelines for adults were released on 15 October 2020 in Canada, though evidence of their associations with health indicators in young adults is sparse. This study aimed to report the prevalence of meeting the 24-h movement guidelines and associations with depressive symptoms in a sample of Chinese university students.

**Methods:**

Cross-sectional data from 1,793 Chinese university students (mean age = 20.7 years, 63.6% female) were used. Sociodemographic information, movement behaviors (physical activity, sedentary behavior, and sleep duration), and depressive symptoms were collected using a self-reported questionnaire.

**Results:**

The prevalence of meeting the 24-h movement guidelines was 27.8% in Chinese university students. Logistic regression results show that compared to those who met the 24-h movement guidelines, odds ratio (OR) for depressive symptoms in those who met fewer recommendations contained in the 24-h movement guidelines were significantly higher (OR for none = 3.4, 95% CI [2.1–5.5], *p* < 0.001; OR for one = 2.7, 95% CI [2.0–3.8], *p* < 0.001; OR for two = 1.5, 95% CI [1.1–2.1], *p* = 0.013).

**Conclusion:**

The prevalence of meeting the 24-h movement guidelines in Chinese university students was relatively low and should be enhanced through multiple strategies. Meeting the 24-h movement guidelines was associated with lower risk for depression in Chinese young adults. It is suggested that moving more, sitting less and sleeping well in this population may reduce the occurrence of depression.

## Introduction

Depression is one of the most prevalent mental health issues with persistent feeling of sadness, tiredness, and anhedonia (*i.e.,* lack of interest or happiness) (World Health Organization, 2020). Depressive symptoms were reported to be associated with substance abuse, eating disorders, dropout, and self-injury among university students (De Aquino Nunes et al., 2024). Moreover, individuals who experienced deterioration in this symptom were associated with greater likelihood of suicide (Eberhard & Weiller, 2016), and exerts a huge health and economic burden on society (Greenberg et al., 2003; Whiteford et al., 2013). During the transition from adolescence to adulthood, university students face multidimensional challenges and pressures (Greenberg et al., 2003; Whiteford et al., 2013), making them more susceptible to psychological problems, including depressive symptoms. Data demonstrates approximately one-third of university students facing mild to severe depressive symptoms (Garlow et al., 2008; Lei et al., 2016; El Ansari et al., 2011). A meta-analysis study in 2016 showed that 23.8% of Chinese university students suffered from depression (Lei et al., 2016). This increase has been attributed to various pandemic-related stressors, including social distancing measures, disruption of routine activities, and concerns about personal health and the economy. Collectively, these data suggest that depressive symptoms are a serious public health problem in university students, which should be addressed by effective measures. Therefore, identifying protective factors against depressive symptoms in this age-group population is urgently needed.

Numerous studies have indicated that movement behaviors, including physical activity (PA), sedentary behavior (SB), and sleep are independent correlates of depressive symptoms in university students. For example, previous studies indicate that regular PA was significantly associated with reduced risk for depressive symptoms in university students (El Ansari et al., 2011; Feng et al., 2014; Vickers et al., 2004; Harbour et al., 2008). Excessive SB has been recognized as a risk factor of developing depressive symptom among university students (Xu et al., 2020; Lee & Kim, 2019). Further, insufficient sleep is also linked to greater odds of being depressed (Feng et al., 2014; Carskadon et al., 2012). As these behaviors are independent factors of depressive symptoms in university students, it is likely that increasing PA, reducing SB and encouraging appropriate sleep concurrently would be beneficial for reducing depressive symptoms. However, it still remains elusive on the combined effects of sufficient PA, limited screen time (ST) and appropriate sleep duration on depressive symptoms.

Since daily time in PA, SB, and sleep are codependent, they should be considered concurrently (Dumuid et al., 2020; Pedisic, 2014; Pedisic, Dumuid & Olds, 2017), making such analyses conducive to informing efficient mental health interventions (Sampasa-Kanyinga et al., 2020). Consequently, in October 2020, the Canadian 24-h movement guidelines for adults were released (Ross et al., 2020) as the world’s first 24-h movement guidelines for adults. The guidelines recommend specific time durations for PA, SB, and sleep in adults (Ross et al., 2020), which offers a new research paradigm for better understanding 24-h movement behaviors and health. Specifically, the guidelines recommend that adults should accumulate at least 150 min of moderate to vigorous PA, limit SB no more than 8 h per day, and maintain 7 to 9 h of good-quality sleep (Ross et al., 2020). However, owing to their novelty, more studies using the guidelines are needed to examine their relationship with different health outcomes, such as depression, and help refine the guidelines in the future.

Although the guidelines are built on rigorous methodology and reliable evidence (Ross et al., 2020; Chaput et al., 2020; Kho et al., 2020; Saunders et al., 2020), little is known about the prevalence of meeting the 24-h movement guidelines for adults and the association with health outcomes. For the 24-h movement guidelines for children and youth, a large number of studies have indicated that meeting more recommendations contained within the guidelines is associated with better mental health in children and adolescents (Janssen, Roberts & Thompson, 2017; Carson et al., 2017; Lee et al., 2018). However, research is needed to examine whether adults who meet the 24-h movement guidelines are also more likely to demonstrate better mental health. Further, a recent review indicated that there was less evidence examining 24-h movement guidelines and mental health indicators in different populations (Rollo, Antsygina & Tremblay, 2020). Examining the associations between the 24-h movement guidelines for adults and health is beneficial to the guidelines’ further development and adoptions in a wider range of populations.

Thus, to better understand the relationship between 24-h movement guidelines for adults and mental health in university students in China (young adults), this study aimed to explore the prevalence of meeting 24-h movement guidelines and the association with depressive symptoms.

## Methods

### Procedure and data collection

This study was a cross-sectional study (online survey), which was based on a longitudinal study (ongoing) to assess the impacts of the coronavirus disease (COVID-19) outbreak on university students’ mental health (Chi et al., 2020). We intended to conduct at least four consecutive surveys (starting on February 2020 and performing the surveys at three-month intervals; all of the four waves of data have been collected). In this study, data from one randomly selected (wave 3, August 2020) were used because of the data availability. A convenient sampling method was adopted in the recruitment of participants in all the waves. Participants from 10 universities in Guang-dong Province were recruited in wave 3 (*n* = 1, 873). For each university, we contacted one staff (some authors’ colleagues) who enabled distribution of our online survey to at least five classes (regardless of majors and grades). Participants (*n* = 1,159) completed and returned the self-report questionnaire with valid data pertaining to the study variables. To increase the sample size, we also invited an additional 714 study participants from another three universities in Anhui Province and 634 completed the questionnaire. Of all the study participants, 1,793 (general response rate = 95.7%; 1,159 from universities in Guangdong and 634 from universities in Anhui) provided answers pertaining to all the variables included in this study. Study participants provided online written consent to participate in this study prior to data collection.

The survey was conducted online (https://www.wjx.cn/jq/88518635.aspx, accessed on 20/2/2021) for both convenience and safety reasons in light of the post-COVID-19 circumstance (field surveys banned for safety considerations). Both of the surveys were conducted in August (21–31st) 2020, when COVID-19 was under control by Chinese government, and students were about to go back to school for fall semester. Over a period of 10 days, students were invited to participate in the survey *via* Tencent’s QQ, WeChat, Weibo, and college-related websites (such as university association websites and bulletin board forums). Participants who had completed all questionnaires (approximately 15 min) were given 10 RMB (Chinese currency) *via* online payment (equivalent to 1.5 USD). The entire study, including recruitment, data collection, and other research procedures, were approved by the Human Research Ethics Committee (No: 2020005) of Shenzhen University.

### Measures

#### Depressive symptoms (PHQ-9)

Participants’ depressive symptoms were assessed by the Patient Health Questionnaire-9 (PHQ-9) consisting of nine items assessing self-report major depressive symptoms relative to the last fortnight where participants responded from 0 = not at all to 3 = almost every day (Kroenke, Spitzer & Williams, 2001). Previous research found that the reliability (internal consistency reliability = 0.86; test-retest reliability = 0.86) and validity (concurrent validity = 0.29; construct validity = 0.89 (Kaiser–Meyer–Olkin measure); criterion validity = 0.92 (area under the curve using receiver operating characteristic)) of PHQ-9 (Chinese version) in university students was acceptable (Du et al., 2017) for use in epidemiological surveys. Previous studies showed acceptable sensitivity and specificity for major depressive symptoms with the PHQ-9 score ≥10 so we used a binary variable (PHQ-9 ≥ 10) for probable depressive symptoms incidence (Du et al., 2017). Binary categorizations of the PHQ-9 were used in statistical analyses.

#### Movement behaviors

Movement behaviors including PA, SB, and sleep were assessed *via* two self-report questionnaires, the International Physical Activity Questionnaire-Short Form (IPAQ-SF) and Pittsburgh Sleep Quality Index (PSQI). The reliability and validity of the IPAQ-SF have been reported in Chinese populations (Macfarlane et al., 2007), indicating the questionnaire was acceptable to capture Chinese adults’ PA (intraclass correlation coefficient = 0.79; concurrent validity = 0.28) and SB (intraclass correlation coefficient = 0.97; concurrent validity = 0.27). In brief, study participants were required to recall their PA (vigorous, moderate, and light) and SB (sitting during leisure and transportation) over the past 7 days. Data processing for variables of PA and SB was consistent with the published guidelines by the IPAQ expert group (International Physical Activity Questionnaire Research Committee, 2005). Study participants’ sleep (hours) was assessed by the PSQI that has been validated in Chinese populations (Guo et al., 2016). Based on the Canadian 24-h movement guidelines for adults (Ross et al., 2020), study participants who reported accumulating ≥150 min per week of moderate- to vigorous-intensity PA (MVPA), ≤8 h per day for SB, and 7–9 h per day for sleep were regarded as meeting the 24-h movement guidelines.

#### Confounding variables

Based on previously published studies (Bennie et al., 2019; Bennie, Teychenne & Tittlbach, 2020), confounding variables included sex (male or female), age (years), body mass index (BMI; calculated from self-reported height (cm) and weight (kg)), siblings (single or two or more), residence (urban or rural), family structure (living with both parents, parents divorced, or other), parents’ educational level (middle school or below, high school, college or university, master or above), number of friends (none, 1–2, 3–5, 6 or more; in our context, friend refers to a person whom one knows and with whom one has a bond of mutual affection, typically exclusive of sexual or family relations), and perceived family affluence using a scale (from 0 to 10) with higher scores indicating higher perceived family affluence (Macfarlane et al., 2007).

### Statistical analyses

Statistical analyses were performed using STATA 16.1 (Stata Corp, College Station, Texas). We set the effect size at 2.2 (odd ratio) according to the previous study, alpha level at 0.05, and power at 0.80, leading to a required minimum sample size of 264 participants. This ensures our study is sufficiently powered to detect meaningful effects. First, descriptive statistics were determined to report frequency (percentage) and mean (standard deviation) of categorical and continuous variables, respectively. Differences in categorical and continuous variables across different groups were examined by chi square test and student t tests, respectively. Correlation coefficients among study variables were determined using Pearson (continuous variables) or Spearman correlations (categorical). To explore the association between combinations (number of recommendations met) of 24-h movement guidelines (meeting the 24-h movement guidelines as reference group) and depressive symptoms, a logistic regression model were developed. A collinearity test was performed to check whether independent variables were affected by multi-collinearity, with variance inflation factor (VIF) <10 suggested as acceptable results (Hair et al., 2009). For the model, depressive symptoms was treated as a binary variable (10-points of PHQ-9 as a threshold). A binary logistic regression with maximum likelihood estimation was used to assess the association between combinations of 24-h movement guidelines and depressive symptoms. Odds ratios (OR) with 95% confidence interval (CI) were reported. Statistical significance was set as *p* < 0.05 (two sided).

## Results

Descriptive characteristics of study participants are presented in Table 1. The mean age of study participants was 20.7 ± 1.6 years. Approximately half of study participants reported meeting the PA guidelines. The prevalence meeting PA guidelines in males was 59.6% compared to 42.5% for females (*p* < 0.001). Over 70.6% reported meeting the SB guidelines (74.9% for males and 68.2% for females, *p* = 0.003). In total, 70.3% met the sleep guidelines without sex difference (male: 70.6%, female: 70.3%, *p* = 0.851). The prevalence of meeting all the three 24-h movement guidelines was 27.8% (higher in males: 37.8% than females: 22.0%, *p* < 0.001), while most study participants met one or two recommendations contained in the guidelines. Study participants reported a mean score of 6.8 ± 5.2 for depressive symptoms and males reported lower scores than did females (males: 6.0 *vs.* female: 7.2, *p* < 0.001). Overall, 23.2% of study participants were categorized as depressed with fewer males than females (*p* = 0.001). More detailed statistics can be found at Table 1.

**Table 1:** Descriptive characteristics of study sample.

	**Total**		**Male**		**Female**		***t or chi Square***	***df***	***p***
	**n**	**%**	**n**	**%**	**n**	**%**			
**Sample**	1,793	100	653	36.4	1,140	63.6			/
**Age (years)**	20.7 ± 1.6		20.8 ± 1.6		20.6 ± 1.6		1.59	1,791	0.110
**BMI (kg/m**^**2**^)	20.3 ± 2.9		21.4 ± 3.1		19.6 ± 2.5		12.94	1,118	0.000
**Siblings**									
Single	613	34.2	277	42.4	336	29.5	30.93	1	0.000
Two or more	1,180	65.8	376	57.6	804	70.5			
**Residence**									
Urban	1,241	69.2	430	65.9	811	71.1	18.29	1	0.000
Rural	552	30.8	223	34.6	329	28.9			
**Family structure**									
Living with both parents	1,261	90.4	593	90.8	1,028	90.2	0.30	2	0.600
Parents divorced	109	6.1	39	6.0	70	6.1			
Other	63	3.5	21	3.	42	3.7			
**Perceived family affluence**	5.7 ± 1.6		5.5 ± 1.7		5.9 ± 1.6		−4.93	1,287	0.000
Father education level									
Middle school or below	867	48.4	328	50.2	539	47.3	6.17	3	0.291
High school	612	34.1	221	33.8	391	34.3			
College or university	250	13.9	76	11.6	174	15.3			
Master or above	64	3.6	28	4.3	36	3.2			
**Mother education level**									
Middle school or below	1,055	58.8	403	61.7	652	57.2	6.07	3	0.245
High school	544	30.3	182	27.9	362	31.8			
College or university	152	8.5	49	7.5	103	9.0			
Master or above	42	2.3	19	2.9	23	2.0			
**Number of friends**									
None	29	1.6	10	1.5	19	1.7	8.68	3	0.052
1–2	590	32.9	209	32.0	381	33.4			
3–5	936	52.2	327	50.1	609	53.4			
6 or more	238	13.3	107	16.4	131	11.5			
**MVPA (min/week)**	127.0 ± 121.0		152.8 ± 132.2		112.2 ± 110.0		9.52	1,050	0.000
**Sedentary time (hours/day)**	6.4 ± 3.7		6.0 ± 3.7		5.0 ± 3.7		−3.51	1,791	0.000
**Sleep duration (hours/day)**	7.4 ± 1.5		7.3 ± 1.6		7.5 ± 1.5		−2.52	1,791	0.012
**Meeting the physical activity guidelines**									
No	921	51.4	266	40.7	655	57.5	46.47	1	0.000
Yes	872	48.6	387	59.3	485	42.5			
**Meeting the sedentary behavior guidelines**									
No	527	29.4	164	25.1	363	31.8	9.05	1	0.003
Yes	1,266	70.6	489	74.9	777	68.2			
**Meeting the sleep guidelines**									
No	532	29.7	192	29.4	340	29.8	0.03	1	0.851
Yes	1,261	70.3	461	70.6	800	70.2			
Positive	416	23.2	123	18.8	293	25.7			
**Combinations of 24-h movement behavior guidelines**									
None	109	6.01	29	4.4	80	7.0	54.27	3	0.000
One	467	26.1	158	24.2	309	27.1			
Two	719	40.1	219	33.5	500	43.9			
All	498	27.8	247	37.8	251	22.0			
**Depressive symptoms**	6.8 ± 5.2		6.0 ± 5.1		7.2 ± 5.2		−4.79	1,791	0.000
**Depressive symptoms (binary)**									
Negative	1,377	76.8	530	81.2	847	74.3	10.98	1	0.001
Positive	416	23.2	123	18.8	293	25.7			

**Notes.**

sd Standard deviation df Degree of freedom MVPA Moderate to vigorous physical activity

Table 2 shows the bivariate correlation coefficients among the variables included in this study. Statistically significant relationships were found between meeting 24-h movement guidelines and depressive symptoms (as a binary variable; *r* = −0.2, *p* < 0.001; see Table 2).

**Table 2:** Correlation matrix among the variables included in this study.

	**1**	**2**	**3**	**4**	**5**	**6**	**7**	**8**	**9**	**10**	**11**	**12**
**1**	1											
**2**	0.0	1										
**3**	0.1^***^	−0.1^***^	1									
**4**	−0.3^***^	0.1^***^	0.0	1								
**5**	0.1^***^	0.0	0.1^*^	−0.1^***^	1							
**6**	−0.1^*^	0.1^***^	−0.2^***^	0.0	0.2^***^	1						
**7**	0.0	0.0	−0.1^***^	0.0	−0.1^***^	0.1^*^	1					
**8**	0.0	−0.1^***^	0.2^***^	0.0	−0.3^***^	−0.3^***^	0.0	1				
**9**	0.0	−0.2^***^	0.2^***^	0.0	−0.3^***^	−0.5^***^	0.0	0.6^***^	1			
**10**	−0.1	0.0	0.1^***^	0.0	−0.0	0.0	0.0	0.0	0.1^**^	1		
**11**	−0.1^***^	0.0	0.1^***^	0.1^***^	0.0	0.0	0.0	0.0	0.0	0.1^**^	1	
**12**	0.1^**^	0.0	−0.1^***^	0.0	0.1^*^	0.0	0.0	0.0	0.0	−0.2^***^	−0.2^***^	1

**Notes.**

* *p* < 0.05.

** *p* < 0.01.

*** *p* < 0.001.

1 Sex.

2 Age.

3 Perceived family affluence.

4 BMI; body mass index.

5 Siblings.

6 Residence.

7 Family structure.

8 Father education level.

9 Mother education level.

10 Number of friends.

11 Combinations of the Canadian 24-h Movement Guidelines for Adults.

12 Depressive symptoms binary.

**Table 3:** The association between meeting 24-h guidelines and depressive symptoms screened positive by generalized liner models.

	**Overall**			**Male**			**Female**		
	**OR**	**95% CI**		**OR**	**95% CI**		**OR**	**95% CI**	
**Meeting none**	3.4	2.1	5.5	3.6	1.4	9.1	3.4	1.9	6.0
**Meeting one**	2.7	2.0	3.8	2.6	1.4	4.6	2.7	1.8	4.1
**Meeting two**	1.5	1.1	2.1	2.2	1.3	3.9	1.3	0.8	1.9

**Notes.**

Models controlled for sex, age, siblings, residence, family structure, father education level, mother education level, perceived family affluence, number of friends, body mass index except for age-specific models. Notes: Two = meeting two recommendations of the 24-hour movement guidelines, One = meeting one recommendation of the 24-hour movement behaviors, None = meeting none of the 24-hour movement behaviors. 24 h movement behaviors, None = meeting none of the 24-hour movement behaviors.

Generalized linear models were performed to determine the association between meeting 24-h movement guidelines and depressive symptoms (as binary variable) after adjusting for the other variables, as shown in Table 3. All VIFs results were below 1.5, indicating the absence of multicollinearity. Compared with meeting the 24-h movement guidelines (meeting all the recommendations), study respondents who met fewer recommendations were more likely to report depressive symptoms. Specifically, those categorized as meeting none of the recommendations, one recommendation, or two recommendations had a higher OR for depressive symptoms in a dose–response gradient (OR for none = 3.4, 95% CI [2.1–5.5], *p* < 0.001; OR for one = 2.7, 95% CI [2.0–3.8], *p* < 0.001; OR for two = 1.5, 95% CI [1.1–2.1], *p* = 0.013). Sex-specific results are also presented in Table 3. In detail, meeting more guidelines was negatively associated with depressive symptoms in males (OR for none = 3.5, 95% CI [1.4–9.1], *p* = 0.012; OR for one = 2.6, 95% CI [1.4–4.6], *p* = 0.003; OR for two = 2.2, 95% CI [1.3–3.9], *p* = 0.008). Such association was partially observed in females but meeting two was not associated with depressive symptoms (OR = 1.3, 95% CI [0.8–1.9], *p* = 0.280).

## Discussion

This study reported the prevalence of meeting the newly released Canadian 24-h movement guidelines for adults and assessed the associations between the different combinations of components of the Canadian 24-h movement guidelines for adults and depressive symptoms (assessed by PHQ-9) in a sample of university students in China. We found that <30% of Chinese young adults met the Canadian 24-h movement guidelines and meeting the guidelines was associated with lower risks for depressive symptoms.

To our knowledge, this study is one of the first to investigate the prevalence of meeting the Canadian 24-h movement guidelines for adults in any jurisdiction, and may help advance our understanding of the relationships between movement behaviors and health in adults. The current study found that 27.8% of Chinese young adults met the 24-h movement guidelines. Owing to limited studies in this field, it is not yet possible to compare our prevalence results with other studies. However, when comparing the prevalence with that in younger populations (children or adolescents), our study showed that Chinese young adults (at least university students) were more likely to exhibit desirable movement behaviors (a higher prevalence of meeting the 24-h movement guidelines). Specifically, for Chinese children and adolescents, a nationally representative study found that only about 5% of them met the age-appropriate 24-h movement guidelines (Chen et al., 2021), which is lower that our result. Another study based on American children and adolescents also showed that the prevalence meeting the 24-h movement guidelines was about 5% (Knell et al., 2019). Some studies reported lower prevalence of meeting the 24-h movement guidelines in children and adolescents (Lee et al., 2019; Manyanga et al., 2019; Shi et al., 2020). Thus, while more Chinese young adults report desirable movement behaviors compared to younger populations, the proportion of meeting these minimal guidelines remains unacceptably low. It should also be noted that the large difference between young adults and children and adolescents might result from survey methodology and survey timing. Concerning the measurements, in our study, IPAQ and PSQI were used to assess movement behaviors while other studies based on children and adolescents used the Health Behavior in School-aged Children (HBSC) questionnaire (Chen et al., 2021; Chen & Yan, 2020) or accelerometers to collect data of MVPA, SB, and sleep (Shi et al., 2020; Roman-Viñas et al., 2016). The difference in the measurements could be explain by the difference in prevalence between young adults, and children and adolescents. Another explanation involves the survey timing. Data collection for the current study was conducted in August, summertime for university students, when they may be more likely to engage in more PA and sleep longer because of less academic pressure and loads. This would in turn lead to limited time spent in SB, resulting in a higher prevalence meeting the 24 movement guidelines in university students compared with children and adolescents who participated in the survey during academic semester (Chen et al., 2021). Of note, it should be mentioned that owing to COVID-19 preventive policies, some study participants’ PA, like outdoor time or leisure activities may be affected. In this regard, our study might be biased to reflect study participant’ regular movement behavior. Future studies are encouraged to monitor 24-h movement behaviors throughout a longer period and across the year to examine the stability of 24-h movement behaviors.

When looking at the prevalence meeting the PA, SB, and sleep recommendations individually, the prevalence meeting the PA recommendation was the lowest in the present study and consequently had the greatest impact on the prevalence of meeting the 24-h movement guidelines. This finding is similar to some studies on children and adolescents (Chen et al., 2020; Shi et al., 2020; Roman-Viñas et al., 2016). Young adults, especially university students, often achieve insufficient MVPA (Zurita-Ortega et al., 2018; Staiano et al., 2018; Unick et al., 2017), which has been well documented in epidemiological surveys. Based on the current study, the promotion of habitual MVPA for Chinese young adults’ will be likely help achieve desired 24-h movement behaviors. More research is also required to understand young adults’ time-use pattern and determine the main determinants of the low prevalence of meeting the 24-h movement guidelines (Chen et al., 2021; Chaput et al., 2014). More time-use epidemiological studies in young adults are needed (Dumuid et al., 2020; Pedisic, Dumuid & Olds, 2017).

Many previously published studies have suggested that meeting the 24-h movement guidelines is associated with better health outcomes, including mental health indicators in young populations (Janssen, Roberts & Thompson, 2017; Lee et al., 2018; Carson et al., 2019). Our study, as one of the very first to assess the 24-h movement guidelines and mental health in young adults, adds evidence on the association between 24-h movement guidelines and depressive symptoms in young adults, which could inform strategies to promote mental health. The present study found that meeting the 24-h movement guidelines presented the lowest risks for depressive symptoms in university students compared to meeting fewer recommendations of 24-h movement guidelines. To the best of our knowledge, there is no comparable research evidence on adults to support or negate our findings. However, evidence from research based on younger populations does provide support. For example, studies by Carson et al. (2017) and Janssen, Roberts & Thompson (2017) indicated that meeting more recommendations contained within 24-h movement guidelines were associated with better mental health in children and adolescents. In US adolescents, meeting the 24-h movement guidelines was significantly associated with reduced depressive symptoms (Zhu, Haegele & Healy, 2019). Similar research findings were also found in a study consisting of South Korean adolescents (Lee et al., 2018). Owing to the limited evidence based on studies of adults, in part because the 24-h guidelines for adults are very new, more studies are encouraged to explore the association between 24-h movement guidelines and depressive symptoms, and other health outcomes, in adults of varying ages. In addition to this, a large body of evidence has suggested that sufficient MVPA (El Ansari et al., 2011; Feng et al., 2014; Vickers et al., 2004; Harbour et al., 2008), limited SB (Xu et al., 2020; Lee & Kim, 2019) and appropriate sleep (Feng et al., 2014; Carskadon et al., 2012) were separate correlates of depressive symptoms. Hence, it is reasonable to expect that their combinations would lead to cumulative effects on reducing depressive symptoms. Evidence from studies using compositional data analysis provide further support to our findings (Dumuid et al., 2020; Zhu, Haegele & Healy, 2019). Studies based on adults have indicated that replacing SB with PA or sleep would be associated with positive mental health outcomes (Larisch et al., 2020; Kitano et al., 2020). This suggests that people with optimal time reallocation for compositions of movement behaviors could gain health benefits. Comparatively, meeting the 24-h movement guidelines could be viewed as a desirable status to achieve to be associated with better mental health outcomes. However, the underlying mechanism(s) linking 24-h movement behaviors to depressive symptoms should be clarified in the future.

An interesting finding is that the association between 24-h movement guidelines and depressive symptoms varied by sex, as in females, meeting any two of the 24-h movement guidelines does not show significantly higher lower odds for depressive symptoms compared to meeting all three guidelines. However, this finding was not found in males. This difference could not be explained in our study, as the mechanism interpreting the sex difference remains unclear. It is, therefore, to explore the sex difference in the association in the future. Of note, this research finding is beneficial to design sex-specific interventions aiming at reducing depression symptoms while through movement behavior changes.

Based on research findings of the current study, some practical implications can be proposed. To prevent mental disorders (*e.g.*, depressive symptoms) of university students, encouragement for more engagement in PA, restrictions of SB, and appropriate sleep would be a beneficial approach. It is needed and recommended to consider the integration of PA, SB, and sleep when designing interventions aiming to reduce or prevent depressive symptoms in university students —a “whole day matters” approach. For university students, schools and families are the two settings in which they spend most of their time, it is therefore recommended to design effective strategies linked with schools and families. However, it should be encouraged to design sex-specific strategies for young adults with different sexes.

Although this study is one of the first to assess the prevalence of meeting the 24-h movement guidelines in young adults and the association between 24-h movement guidelines and depressive symptoms, some inherent study limitations should be mentioned. First, owing to the cross-sectional nature of the study, we cannot infer causality for the association between 24-h movement guidelines and depressive symptoms. Second, self-reported questionnaires were used to assess 24-h movement behaviors in our study, which could result in measurement errors. Third, our study recruited samples using a convenient sampling procedure instead of random sampling, which reduces the representativeness; so, the research findings may not be generalizable into other similar populations. Fourth, the Canadian 24-h guidelines also recommend that within the SB period, recreational screen time should not exceed three hours. Future research should also incorporate this recommendation. Fifth, there are many factors related with depressive symptoms in university students. However, in our study, only a limited number of confounding variables were included. Sixth, for the measures of family structure, we failed to collect more details and information on it, which may result in some bias in estimating the association between independents and dependents. Seventh, our study’s region targeted a few provinces, which limits the generalizability of our findings to all university students to the whole China. Finally, owing to study survey period was during the COVID-19 pandemic, some factors would have influences on 24-h movement behaviors and depressive symptoms, which may not be considered in our study. Such omissions could result in bias to our study results. It should be, therefore, extremely cautious to interpret our results.

## Conclusions

This study reports the prevalence of meeting the 24-h movement guidelines for adults and assessed the association with depressive symptoms in university students (young adults). We observed that in this sample less than 30% of Chinese university students met the 24-h movement guidelines and that meeting more recommendations within the guidelines had lower odds for depressive symptoms. The current study provides preliminary evidence to support the concurrence of sufficient PA, limited SB, and appropriate sleep associated with better mental health. For university students’ mental health, it is recommended to make them move more, sit less and sleep well. Further research is recommended to confirm these findings and to explore associations between adult 24-h movement behaviors and other mental health outcomes.

## Supplemental Information

10.7717/peerj.17217/supp-1 Supplemental Information 1 24-hour and Depression Datasets

10.7717/peerj.17217/supp-2 Supplemental Information 2 Codebook

## Additional Information and Declarations

### Data Availability

The following information was supplied regarding data availability: The raw measurements are available in the Supplementary File.

## References

[ref-1] Bennie JA, De Cocker K, Biddle SJH, Teychenne MJ (2019). Joint and dose-dependent associations between aerobic and muscle-strengthening activity with depression: a cross-sectional study of 1.48 million adults between 2011 and 2017. Depression and Anxiety.

[ref-2] Bennie JA, Teychenne M, Tittlbach S (2020). Muscle-strengthening exercise and depressive symptom severity among a nationally representative sample of 23635 German adults. Journal of Affective Disorders.

[ref-3] Carskadon MA, Sharkey KM, Knopik VS, McGeary J.E (2012). Short sleep as an environmental exposure: a preliminary study associating 5-HTTLPR genotype to self-reported sleep duration and depressed mood in first-year university students. Sleep.

[ref-4] Carson V, Chaput J-P, Janssen I, Tremblay MS (2017). Health associations with meeting new 24-hour movement guidelines for Canadian children and youth. Preventive Medicine.

[ref-5] Carson V, Ezeugwu VE, Tamana SK, Chikuma J, Lefebvre DL, Azad MB, Moraes TJ, Subbarao P, Becker AB, Turvey SE, Sears MR, Mandhane PJ (2019). Associations between meeting the Canadian 24-hour movement guidelines for the early years and behavioral and emotional problems among 3-year-olds. Journal of Science and Medicine in Sport.

[ref-6] Chaput J-P, Carson V, Gray CE, Tremblay MS (2014). Importance of all movement behaviors in a 24 hour period for overall health. International Journal of Environmental Research and Public Health.

[ref-7] Chaput J-P, Dutil C, Featherstone R, Ross R, Giangregorio L, Saunders TJ, Janssen I, Poitras VJ, Kho ME, Ross-White A, Carrier J (2020). Sleep duration and health in adults: an overview of systematic reviews. Applied Physiology, Nutrition, and Metabolism.

[ref-8] Chen S-T, Liu Y, Tremblay MS, Hong J-T, Tang Y, Cao Z-B, Zhuang J, Zhu Z, Wu X, Wang L, Cai Y, Chen P (2021). Meeting 24-hour movement guidelines: prevalence, correlates and the relationships with overweight and obesity among Chinese children and adolescents. Journal of Sport and Health Science.

[ref-9] Chen S-T, Yan J (2020). Prevalence and selected sociodemographic of movement behaviors in schoolchildren from low- and middle-income families in Nanjing, China: a cross-sectional questionnaire survey. Children.

[ref-10] Chi X, Becker B, Yu Q, Willeit P, Jiao C, Huang L, Hossain MM, Grabovac I, Yeung A, Lin J, Veronese N, Wang J, Zhou X, Doig SR, Liu X, Carvalho AF, Yang L, Xiao T, Zou L, Fusar-Poli P, Solmi M (2020). Prevalence and psychosocial correlates of mental health outcomes among chinese college students during the coronavirus disease (COVID-19) pandemic. Frontiers in Psychiatry.

[ref-11] De Aquino Nunes JC, Magalhães DOL, Rodrigues AVS, Saraiva ANU, De Jesus E, Santos CP, De Oliveira EK, Fagundes Souto D, Fonseca Lisboa B, Almeida Nunes T, Azevedo Ledo L (2024). Prevalence of depressive symptoms, anxiety, suicidal ideation and associated factors among higher education students. Health and Society.

[ref-12] Du N, Yu K, Ye Y, Chen S (2017). Validity study of Patient Health Questionnaire-9 items for Internet screening in depression among Chinese university students. Asia-Pacific Psychiatry.

[ref-13] Dumuid D, Pedišić Ž, Palarea-Albaladejo J, Martín-Fernández JA, Hron K, Olds T (2020). Compositional data analysis in time-use epidemiology: what, why, how. International Journal of Environmental Research and Public Health.

[ref-14] Eberhard J, Weiller E (2016). Suicidality and symptoms of anxiety, irritability, and agitation in patients experiencing manic episodes with depressive symptoms: a naturalistic study. Neuropsychiatric Disease and Treatment.

[ref-15] El Ansari W, Stock C, Phillips C, Mabhala A, Stoate M, Adetunji H, Deeny P, John J, Davies S, Parke S, Hu X, Snelgrove S (2011). Does the association between depressive symptomatology and physical activity depend on body image perception? A survey of students from seven universities in the UK. International Journal of Environmental Research and Public Health.

[ref-16] Feng Q, Zhang Q-L, Du Y, Ye Y-L, He Q-Q (2014). Associations of physical activity, screen time with depression, anxiety and sleep quality among chinese college freshmen. PLOS ONE.

[ref-17] Garlow SJ, Rosenberg J, Moore JD, Haas AP, Koestner B, Hendin H, Nemeroff CB (2008). Depression, desperation, and suicidal ideation in college students: results from the American Foundation for Suicide Prevention College Screening Project at Emory University. Depression and Anxiety.

[ref-18] Greenberg PE, Kessler RC, Birnbaum HG, Leong SA, Lowe SW, Berglund PA, Coreylisle PK (2003). The economic burden of depression in the United States: how did it change between 1990 and 2000?. Journal of Clinical Psychiatry.

[ref-19] Guo S, Sun W, Liu C, Wu S (2016). Structural validity of the pittsburgh sleep quality index in chinese undergraduate students. Frontiers in Psychology.

[ref-20] Hair JF, Black WC, Babin BJ, Anderson RE (2009). Multivariate data analysis 7th ed.

[ref-21] Harbour VJ, Behrens TK, Kim HS, Kitchens C.L (2008). Vigorous physical activity and depressive symptoms in college students. Journal of Physical Activity and Health.

[ref-22] International Physical Activity Questionnaire Research Committee (2005). Guidelines for data processing and analysis of the International Physical Activity Questionnaire (IPAQ)-short and long forms. http://www.ipaq.ki.se/scoring.pdf.

[ref-23] Janssen I, Roberts KC, Thompson W (2017). Is adherence to the Canadian 24-Hour movement behaviour guidelines for children and youth associated with improved indicators of physical, mental, and social health?. Applied Physiology, Nutrition, and Metabolism.

[ref-24] Kho ME, Poitras VJ, Janssen I, Chaput J-P, Saunders TJ, Giangregorio LM, Tomasone JR, Ross-White A, Ross R (2020). Development and application of an outcome-centric approach for conducting overviews of reviews. Applied Physiology, Nutrition, and Metabolism.

[ref-25] Kitano N, Kai Y, Jindo T, Tsunoda K, Arao T (2020). Compositional data analysis of 24-hour movement behaviors and mental health in workers. Preventive Medicine Reports.

[ref-26] Knell G, Durand CP, Kohl HW, Wu IHC, Gabriel KP (2019). Prevalence and likelihood of meeting sleep, physical activity, and screen-time guidelines among US youth. JAMA Pediatrics.

[ref-27] Kroenke K, Spitzer RL, Williams JB (2001). The PHQ-9: validity of a brief depression severity measure. Journal of General Internal Medicine.

[ref-28] Larisch L-M, Kallings LV, Hagströmer M, Desai M, Von Rosen P, Blom V (2020). Associations between 24 h movement behavior and mental health in office workers. International Journal of Environmental Research and Public Health.

[ref-29] Lee E-Y, Carson V, Jeon JY, Spence JC, Tremblay MS (2019). Levels and correlates of 24-hour movement behaviors among South Koreans: results from the Korea National Health and Nutrition Examination Surveys, 2014 and 2015. Journal of Sport and Health Science.

[ref-30] Lee E, Kim Y (2019). Effect of university students’ sedentary behavior on stress, anxiety, and depression. Perspectives in Psychiatric Care.

[ref-31] Lee E-Y, Spence JC, Tremblay MS, Carson V (2018). Meeting 24-hour movement guidelines for children and youth and associations with psychological well-being among South Korean adolescents. Mental Health and Physical Activity.

[ref-32] Lei X-Y, Xiao L-M, Liu Y-N, Li Y-M (2016). Prevalence of depression among chinese university students: a meta-analysis. PLOS ONE.

[ref-33] Macfarlane DJ, Lee CC, Ho EY, Chan K, Chan DT (2007). Reliability and validity of the Chinese version of IPAQ (short, last 7 days). Journal of Science and Medicine in Sport.

[ref-34] Manyanga T, Barnes JD, Chaput J-P, Katzmarzyk PT, Prista A, Tremblay MS (2019). Prevalence and correlates of adherence to movement guidelines among urban and rural children in Mozambique: a cross-sectional study. International Journal of Behavioral Nutrition and Physical Activity.

[ref-35] Pedisic Z (2014). Measurement issues and poor adjustments for physical activity and sleep undermine sedentary behaviour research—The focus should shift to the balance between sleep, sedentary behaviour, standing and activity. Kinesiology.

[ref-36] Pedisic Z, Dumuid D, Olds TS (2017). Integrating sleep, sedentary behaviour, and physical activity research in the emerging field of time-use epidemiology: definitions, concepts, statistical methods, theoretical framework, and future directions. Kinesiology.

[ref-37] Rollo S, Antsygina O, Tremblay MS (2020). The whole day matters: understanding 24-hour movement guideline adherence and relationships with health indicators across the lifespan. Journal of Sport and Health Science.

[ref-38] Roman-Viñas B, Chaput J-P, Katzmarzyk PT, Fogelholm M, Lambert EV, Maher C, Maia J, Olds T, Onywera V, for the ISCOLE Research Group (2016). Proportion of children meeting recommendations for 24-hour movement guidelines and associations with adiposity in a 12-country study. International Journal of Behavioral Nutrition and Physical Activity.

[ref-39] Ross R, Chaput J-P, Giangregorio LM, Janssen I, Saunders TJ, Kho ME, Poitras VJ, Tomasone JR, El-Kotob R, McLaughlin EC, Duggan M, Carrier J, Carson V, Chastin SF, Latimer-Cheung AE, Chulak-Bozzer T, Faulkner G, Flood SM, Gazendam MK, Healy GN, Katzmarzyk PT, Kennedy W, Lane KN, Lorbergs A, Maclaren K, Marr S, Powell KE, Rhodes RE, Ross-White A, Welsh F, Willumsen J, Tremblay M.S (2020). Canadian 24-Hour Movement Guidelines for Adults aged 18–64 years and Adults aged 65 years or older: an integration of physical activity, sedentary behaviour, and sleep. Applied Physiology, Nutrition, and Metabolism.

[ref-40] Sampasa-Kanyinga H, Colman I, Goldfield GS, Janssen I, Wang J, Podinic I, Tremblay MS, Saunders TJ, Sampson M, Chaput J-P (2020). Combinations of physical activity, sedentary time, and sleep duration and their associations with depressive symptoms and other mental health problems in children and adolescents: a systematic review. International Journal of Behavioral Nutrition and Physical Activity.

[ref-41] Saunders TJ, McIsaac T, Douillette K, Gaulton N, Hunter S, Rhodes RE, Prince SA, Carson V, Chaput J-P, Chastin S, Giangregorio L, Janssen I, Katzmarzyk PT, Kho ME, Poitras VJ, Powell KE, Ross R, Ross-White A, Tremblay MS, Healy GN (2020). Sedentary behaviour and health in adults: an overview of systematic reviews. Applied Physiology, Nutrition, and Metabolism.

[ref-42] Shi Y, Huang WY, Sit CH-P, Wong SH-S (2020). Compliance with 24-hour movement guidelines in Hong Kong adolescents: associations with weight status. Journal of Physical Activity and Health.

[ref-43] Staiano AE, Martin CK, Champagne CM, Rood JC, Katzmarzyk PT (2018). Sedentary time, physical activity, and adiposity in a longitudinal cohort of nonobese young adults. American Journal of Clinical Nutrition.

[ref-44] Unick JL, Lang W, Tate DF, Bond DS, Espeland MA, Wing R.R (2017). Objective estimates of physical activity and sedentary time among young adults. Journal of Obesity.

[ref-45] Vickers K, Patten C, Bronars C, Lane K, Stevens S, Croghan I, Schroeder D, Clark M (2004). Binge drinking in female college students: the association of physical activity, weight concern, and depressive symptoms. Journal of the American College of Health.

[ref-46] Whiteford HA, Degenhardt L, Rehm J, Baxter AJ, Ferrari AJ, Erskine HE, Charlson FJ, Norman RE, Flaxman AD, Johns N, Burstein R, Murray CJL, Vos T (2013). Global burden of disease attributable to mental and substance use disorders: findings from the Global Burden of Disease Study 2010. Lancet.

[ref-47] World Health Organization (2020). Depression. https://www.who.int/westernpacific/health-topics/depression.

[ref-48] Xu Z, Xu Q, Wang Y, Zhang J, Liu J, Xu F (2020). Association of sedentary behavior and depression among college students majoring in design. International Journal of Environmental Research and Public Health.

[ref-49] Zhu X, Haegele JA, Healy S (2019). Movement and mental health: behavioral correlates of anxiety and depression among children of 6–17 years old in the U.S. Mental Health and Physical Activity.

[ref-50] Zurita-Ortega F, Román-Mata SS, Chacón-Cuberos R, Castro-Sánchez M, Muros JJ (2018). Adherence to the mediterranean diet is associated with physical activity, self-concept and sociodemographic factors in university student. Nutrients.

