# Supplementary material for: Cross-sectional association between 24-hour movement guidelines and depressive symptoms in Chinese university students
Source: PeerJ. 2024 Apr 15;12:e17217. doi: 10.7717/peerj.17217 (PMC11025541; doi:10.7717/peerj.17217)
Supplement: Supplemental Information 2 [file peerj-12-17217-s002.pdf]

| Name | Label | Value | Labels |
|---|---|---|---|
| gender | sex | | |
| | | 1 | male |
| | | 2 | female |
| age | age | | |
| BMI | BMI | | |
| siblings | number of children in the family | | |
| | | 1 | single |
| | | 2 | two or more |
| residence | geographic location | | |
| | | 1 | urban |
| | | 2 | rural |
| fa_structure | family structure | | |
| | | 1 | living with both parents |
| | | 2 | parents divorced |
| | | 3 | other |
| fa_edu | father education | | |
| | | 1 | middle school |
| | | 2 | high school |
| | | 3 | college |
| | | 4 | graduate and aobve |
| mo_edu | mother education | | |
| | | 1 | middle school |
| | | 2 | high school |
| | | 3 | college |
| | | 4 | graduate and aobve |
| friends | number of close friends | | |
| | | 1 | none |
| | | 2 | 1-2 |
| | | 3 | 3-5 |
| | | 4 | 6 or more |
| MVPA_guidelines | meeting the PA guidelines | | |
| | | 0 | not meeting |
| | | 1 | meeting |
| sleep_guidelines | meeting the sleep guidelines | | |
| | | 0 | not meeting |
| | | 1 | meeting |
| SB_guidelines | meeting the SB guidelines | | |
| | | 0 | not meeting |
| | | 1 | meeting |
| co_24 | meeting the PA, SB, and sleep guidelines | | |
| | | 0 | meet none |
| | | 1 | meet one |
| | | 2 | meet two |
| | | 3 | meet all |
| PHQ_cat | depression binary | | |
| | | 0 | negative |
| | | 1 | positive |
